# A Three Factor Model of Parental Coping With Childhood Cancer

**DOI:** 10.1002/pon.70294

**Published:** 2025-10-07

**Authors:** Oz Hamtzani, Michael J. Dolgin, Talma Kushnir

**Affiliations:** ^1^ The Edmond and Lily Safra Children's Hospital Sheba Tel‐HaShomer Ramat Gan Israel; ^2^ Department of Psychology Ariel University Ariel Israel; ^3^ The Adelson School of Medicine Ariel University Ariel Israel

**Keywords:** cancer, childhood cancer, coping flexibility, coping repertoire, coping techniques, oncology, parental distress

## Abstract

**Objectives:**

Parents of children with cancer are at increased risk for anxiety, depression, and post‐traumatic stress, although wide variability among parents has been documented. This cross‐sectional study was designed to examine the individual contributions and simultaneous interaction of three coping constructs—coping strategy, repertoire of coping techniques, and flexibility in applying these techniques—in parental distress related to childhood cancer.

**Methods:**

A sample of 88 mothers and 57 fathers (*N* = 145) of children undergoing active cancer treatment were recruited from a pediatric hematology‐oncology department. Parents' ages ranged from 22 to 58 years. Parents completed standardized measures including the *Brief COPE, Coping Flexibility Scale‐Revised,* the *Pediatric Parenting Stress Inventory*, and the *Profile of Mood States*.

**Results:**

Avoidance‐focused coping strategy, repertoire of coping techniques, and coping flexibility were individually found to be significantly correlated with parental distress. Bootstrap mediation analysis revealed that the collective model explained 37%–43% of the variance in parental distress, with avoidance‐focused coping strategy emerging as the most significant predictor, accounting for approximately 29% of the total variance.

**Conclusions:**

These results suggest that clinical interventions should prioritize identifying and reducing parental reliance on avoidance‐focused coping techniques as a primary target. Subsequently, expanding parents' repertoire of problem‐ and emotion‐focused coping techniques and enhancing flexibility in their application could lead to better distress reduction. However, the cross‐sectional design limits causal interpretation, and future longitudinal studies employing larger sample sizes are needed to establish the relationships between these constructs.

## 1 Introduction

While advances in medical treatment have led to improved medical outcomes, pediatric cancer is still the leading non‐accidental cause of death in children and adolescents [1]. A significant body of literature attests to the psychological challenges facing parents of children diagnosed with cancer, owing to their pivotal role in maintaining personal, dyadic, and family equilibrium as well as in navigating the medical system and meeting treatment demands [2, 3, 4]. Those studies also suggest that parents are at increased risk for anxiety, depression, and post‐traumatic stress symptoms. While the severity of symptoms declines within the first year post‐diagnosis, some parents experience these symptoms even 5 years post‐diagnosis [2, 3]. Still, there is a wide variance among parents in terms of the degrees and trajectories of distress over time [5]. A better understanding of the sources of this variability is central to the identification of risk and resilience factors and to the development of appropriate and effective interventions. In this regard, the focus of the current study is on the interactive impact of coping techniques, coping repertoire, and coping flexibility on parental distress related to childhood cancer. These dimensions have not been systematically examined in parents of children with cancer, despite their being potentially modifiable factors that might be targeted through therapeutic intervention.

## 2 Coping

Coping is defined as efforts to prevent or diminish threat, harm, and loss or to reduce the distress, that is, often associated with those experiences [6]. Various terms are used in the coping literature, and it is necessary to clarify the vocabulary used and to distinguish between five types of coping constructs—coping resources, coping styles, coping methods, coping strategies, and coping techniques. Coping resources are defined as internal and external assets that are available to the individual in meeting the challenges of a stressful situation [7, 8]. Coping style refers to a pattern of behavior that tends to be relatively stable over time and across situations in a person's cognitive and behavioral responses to stressors [9]. Coping method refers to the cognitive‐behavioral approach by which an individual attempts to develop effective solutions or ways of dealing with real‐life problems or stressors [10]. Coping strategies are defined as a set of actions, behaviors, and thoughts that are dictated by the nature of the stressor [6]. Three common coping strategies are identified: Problem‐, emotion‐, and avoidance‐focused coping [8, 11, 12]. Studies have found that in situations where the stressor can be altered, a problem‐focused strategy leads to better psychological outcomes, while in situations where the stressor cannot be altered, an emotion‐focused strategy leads to better outcomes [6, 8]. Finally, based on the person's choice of coping strategy, one may employ specific coping techniques. Problem‐focused coping techniques involve acting on the environment (e.g., seeking instrumental support from others) or on the self (e.g., planning, active coping) to affect and change the nature of stressful situations. Emotion‐focused coping techniques aim to regulate one's emotions (e.g., prayer, relaxation) when the situation cannot be altered.

### 2.1 Avoidance‐Focused Coping Strategy, Coping Repertoire, and Coping Flexibility

Regardless of the nature of the stressor, techniques that aim at avoiding the stressor (e.g., substance use, denial) or at blaming oneself, are typically associated with greater psychological distress. Studies with parents of children with cancer have found avoidance‐focused coping strategy to be correlated with higher levels of depression and anxiety symptoms [13, 14]. Studies suggest that no single coping technique effectively mitigates psychological distress across all types of stressors. Rather, coping techniques should ideally change over time according to the demands of the particular stressor and the available coping resources [15]. Thus, having a greater repertoire of coping techniques enables individuals to select the most appropriate technique for each specific stressor, which may lead to better psychological outcomes compared to relying on fewer, potentially less suitable techniques.

Empirical findings regarding the role of repertoire of coping techniques, however, present a more nuanced picture. While several studies have found that greater repertoire leads to better psychological adjustment [16, 17, 18], others report greater repertoire to be associated with increased psychological distress [19]. One possible reason for this inconsistency may lie in the different approaches used to assess coping repertoire. Heffer & Willoughby [16] quantified coping repertoire based on a count of positive techniques (those associated with decreased psychological distress) and negative techniques (those associated with increased psychological distress). By this method, a repertoire count of positive techniques was negatively correlated with distress. In contrast, Kato's [19] approach counts the total number of coping techniques that an individual employs in response to stress, regardless of their adaptive value. By this approach, greater repertoire of coping techniques was found to be associated with greater psychological distress.

Beyond the mere possession of a broad coping repertoire, it is the flexible application of coping techniques that serves as a key mediator in the coping process [20]. Coping flexibility, the ability to relinquish a coping technique regarded as ineffective and devise and implement an alternative one, has been identified as a critical factor in reducing psychological distress [19]. This definition suggests that effective copers adjust their coping techniques to match the unique characteristics of the stressor they face. Bonanno & Burton [20] proposed a theoretical model outlining three sequential stages in coping: initial appraisals, strategy and techniques selection, and flexible implementation of the chosen techniques. In the first stage, individuals engage in primary (e.g., *How will this affect my well‐being?*) and secondary appraisals (e.g., *What can I do about it?*), in accordance with the transactional theory of stress [8, 15]. Based on these appraisals, individuals then select techniques from their available repertoire. The final stage involves coping flexibility, a process which involves evaluating the effectiveness of the chosen technique, discontinuing it if it proves ineffective, and replacing it with a more appropriate alternative. Empirical findings have shown that higher levels of coping flexibility are associated with improved psychological distress outcomes in parents population [18, 21]. Thus, coping repertoire is an essential precursor to flexibility, as without a diverse set of techniques to draw from, flexibility cannot manifest. Likewise, greater repertoire of techniques requires flexibility in their application to result in more positive adjustment outcomes.

### 2.2 Current Study Rationale and Hypotheses

The current study examined the individual and collective impact of three coping constructs—the use of avoidance‐focused coping strategy, repertoire of coping techniques, and coping flexibility—on parental distress due to their child's cancer, as well as the relationships between these constructs. Understanding how these constructs work together to affect distress levels, which has not been systematically examined in parents of children with cancer, could inform the refinement of existing interventions and guide the development of more targeted therapeutic approaches for this population.

Based on research literature, we hypothesized a mediation model in which a broader repertoire of positive‐neutral coping techniques would be associated with greater coping flexibility, which in turn would lead to reduced parental distress, while controlling for avoidance‐focused coping strategy. Within this model, we expected that the three coping constructs would operate through different pathways: avoidance‐focused coping strategy would demonstrate a direct relationship with parental distress, whereas coping repertoire would influence distress indirectly through its effect on coping flexibility.

## 3 Methods

### 3.1 Participants

Participants were recruited from the population of parents of children diagnosed with cancer in the pediatric hematology‐oncology department of the *Edmond and Lily Safra Children's Hospital* between April 2022 and May 2023. Parents of children with any form of malignancy or central nervous system tumor, and during all phases of active treatment were offered participation. Further eligibility criteria included the ability to speak and read Hebrew, Arabic, or English. Parents were approached no earlier than 6 weeks post‐diagnosis in order to avoid potential burden during the critical, and sometimes chaotic, period following diagnosis and treatment initiation. Additionally, parents whose child was in an acute medical crisis during the recruitment phase were not approached for participation.

In order to determine the required sample size, an a priori power analysis was conducted using *Monte Carlo* simulation in *R* software via the *simsem* package [22]. Based on prior literature and theoretical considerations, we estimated standardized regression coefficients (beta) for direct paths to parental distress as follows: 0.12 for coping repertoire, 0.32 for coping flexibility [23], and 0.50 for avoidance‐focused coping strategy [13]. Additionally, while studies suggest a standardized regression coefficient of 0.48 for the correlation between psychological inflexibility and avoidance‐focused coping strategy [24], we estimated a more conservative standardized effect of 0.15 for the association with coping flexibility. In the absence of prior literature on this specific pathway, we estimated a conservative medium effect size of 0.30 for the path from coping repertoire to coping flexibility, based on theoretical models regarding the relationship between these coping constructs [19, 20]. The analysis targeted at least 80% power with a type 1 error rate (*alpha*) of 5%. The analysis yielded a required sample size of 122 participants. The current study recruited 145 participants, ensuring adequate statistical power.

### 3.2 Measures

#### 3.2.1 Demographic and Medical Data

Parents were asked to provide information regarding their gender, age, place of birth, education level, marital status, number of children, cancer history, and religion. In addition, parents were asked to provide information regarding their ill child, including gender, age, diagnosis, time since diagnosis, and treatment protocol.

#### 3.2.2 Brief COPE

Developed by Carver [25], adapted to Hebrew by Ben‐Zur & Zeidner [26], and to Arabic by Hamdan‐Mansour et al. [27], the *Brief COPE* is a 28‐item self‐report instrument designed to measure the use of specific coping techniques when facing stressful situations. It measures the frequency of use of 14 coping techniques, two items per technique. Respondents are asked to endorse each item using a five‐point scale ranging from “*0*” (*I haven't been doing this at all*) to “*4*” (*I've been doing this a lot*). The scoring is based on the average of the two items that make up each coping technique. In the current study, respondents were asked to rate their use of each coping technique specifically in relation to their child's treatment period. From this instrument, we derived measures of avoidance‐focused coping strategy and coping repertoire as described below.

##### 3.2.2.1 Coping Strategies

Following Endler & Parker's [11] classification, coping techniques were classified into three distinct coping strategies: problem‐focused (i.e., seeking instrumental support, planning, and active coping), emotion‐focused (i.e., self‐distraction, venting, acceptance, humor, religion, positive reframing, and social emotional support), and avoidance‐focused (behavioral disengagement, denial, substance use, and self‐blame). Acceptable reliability was established for the problem‐ (*Cronbach α* = 0.80; *Guttman's λ*_*2*_ = 0.81), emotion‐ (*Cronbach α* = 0.70; *Guttman's λ*_*2*_ = 0.73), and avoidance‐focused (*Cronbach α* = 0.67; *Guttman's λ*_*2*_ = 0.70) coping strategies.

##### 3.2.2.2 Coping Repertoire

Following Heffer and Willoughby's [16] count‐based approach, each coping technique was coded as “*0*” (i.e., not using this technique at all) or “*1*” (i.e., using this technique to any degree). Coping techniques were then counted into two repertoire classifications based on their distress outcomes: those associated with neutral‐to‐positive outcomes (i.e., acceptance, humor, positive reframing, social emotional support, social instrumental support, planning, and active coping), and those associated with negative outcomes (i.e., behavioral disengagement, denial, substance use, self‐blame, self‐distraction, and venting). In the current study, we used the term “*coping repertoire*” to refer to those techniques associated with neutral‐to‐positive outcomes. Religion was not calculated into either positive or negative coping repertoire for methodological reasons which will be addressed in the *Discussion* section.

#### 3.2.3 Coping Flexibility Scale‐Revised (CFS‐R)

Developed by Kato [19], the *CFS‐R* is a 12‐item self‐report instrument measuring coping flexibility via three subscales of four items each: 1. Abandonment (i.e., the ability to relinquish a coping technique recognized as ineffective; e.g., “*I can stop using a failed coping technique”*), 2. Re‐coping (i.e., implementing an alternative coping technique that differs from the one relinquished in the previous process; e.g., “*If I did not cope well, I use an alternative coping technique”*), and; 3. Meta‐coping (i.e., the ability to monitor and provide feedback on the coping process; e.g., “*I cope with stress by establishing clear objectives”*). Respondents are asked to endorse each item on a four‐point scale ranging from “*0*” (*not applicable*) to “*3*” (*very applicable*). A higher score indicates a higher level of coping flexibility. Psychometrically, *CFS‐R* is characterized by good reliability estimates for abandonment (*Cronbach α* = 0.82), re‐coping (*Cronbach α* = 0.84) and meta‐coping (*Cronbach α* = 0.80). The *CFS‐R* was translated to Hebrew and Arabic using standard back‐translation procedures. While the “*Meta‐coping*” subscale was found to be associated with “*Abandonment*” and “*Re‐Coping*” scales, Kato [19] could not provide sufficient information regarding the predictive validity of this subscale. Therefore, a general flexibility scale was calculated based on the *“Abandonment”* and the *“Re‐Coping”* subscales. Higher scores indicate greater coping flexibility.

#### 3.2.4 Pediatric Parenting Stress Inventory (PPSI)

Developed by Devine et al. [28], the *PPSI* is a 35‐item self‐report instrument designed to measure different problems and sources of distress experienced by parents of children with chronic illnesses. Respondents are asked to endorse items on a 5‐point scale ranging from “*0*” (*not at all*) to “*4*” (*to a very large extent*), indicating how much each item was a problem for them during the past week. The questionnaire produces a general score (*Cronbach α* = 0.92) as well as subscores in four different areas in which parents of children with chronic illness typically report problems: (1). Managing the ill child's needs (e.g., *communication with medical staff*; *Cronbach α* = 0.85); (2). Emotional and physical problems of the parent (e.g., *anxiety and depression*; *Cronbach α* = 0.94); (3.) Managing finances (e.g., *reduction in income*; *Cronbach α* = 0.74); 4. Managing family life (e.g., *arrangements for the healthy siblings*; *Cronbach α* = 0.84). The emotional and physical problem subscale was found to correlate highly with overall mood, depression, and posttraumatic stress symptoms [28]. The *PPSI* was translated to Arabic using standard back‐translation procedures.

#### 3.2.5 Profile of Mood State Short Form (POMS‐SF)

Developed by Shacham [29], adapted to Hebrew by Netz et al. [30] and to Arabic by Aroian et al. [31], the *POMS‐SF* is a 28‐item self‐report instrument designed to measure affective mood states experienced over the previous week in five dimensions: (1) *Anger‐Hostility*; (2) *Depression‐Dejection*; (3) *Fatigue‐Inertia*; (4) *Tension‐Anxiety*, and; (5) *Vigor‐Activity*. Respondents are asked to endorse each item using a five‐point scale ranging from “*0*” (*not at all*) to “*4*” (*to a very large extent*). The measure produces a general mood score, as well as subscores in each of the five mood dimensions (*Cronbach α* = 0.91). For the purposes of the current study, the general mood score was used. Higher scores indicate greater parental distress.

### 3.3 Procedure

Eligible participants were contacted by the first author at the pediatric hematology‐oncology department, offered participation, and asked to provide informed consent. Measures were administered face‐to‐face or electronically, based on the participant's preference. A modest incentive in the form off a 100 *NIS* gift certificate was granted as compensation for participants' time and effort.

### 3.4 Data Analyses

Data analyses progressed through three phases: descriptive statistics, bivariate correlations between coping and distress measures, and mediation analysis to test the hypothesis that broader coping repertoire would enable greater coping flexibility, which would lead to less parental distress, while controlling for avoidance‐focused coping strategy. Bootstrap mediation analysis (5000 resamples) was conducted using the *PROCESS* macro for *R* [32].

## 4 Results

### 4.1 Participants

Out of 151 eligible parents who were approached to participate in the study, 145 parents consented to participate and completed data collection. Table 1 displays parental demographic data. The study sample included 61% mothers and 39% fathers. Parents' age range was 22–58 years. The majority of parents were married or in a relationship. Table 2 displays the ill child's demographic and medical data. These included equal proportions of male and female patients [*χ* ^*2*^ *(1)* = 0.34, *p* = 0.561] with an age range of 0–21 years.

**TABLE 1:** Parents' demographic data.

Measure	*n* (%)
Gender	
Mothers	88 (60.7%)
Fathers	57 (39.3%)
Age, M (SD)	40.2 (7.9)
Marital status	
Married/in a relationship	127 (87.6%)
Not in a relationship	18 (12.4%)
Religion	
Jewish	88 (60.7%)
Moslem	53 (36.5%)
Christian	4 (2.8%)
Cancer history	
No	85 (59.9%)
Yes	57 (40.1%)
Education	
High school education	37 (26.1%)
High school diploma	25 (17.6%)
Bachelor's degree/professional certificate	47 (33.1%)
Master's degree or higher	33 (23.2%)
Number of children	
1	10 (6.9%)
2	25 (17.2%)
3	40 (27.6%)
4+	70 (48.3%)

**TABLE 2:** Child‐Patient demographic and medical data.

Measure	*n* (%)
Gender	
Female	69 (47.6%)
Male	76 (52.4%)
Age, M (SD)	9.5 (5.1)
Diagnosis	
CNS	22 (15.2%)
Leukemia	52 (35.9%)
Lymphoma	8 (5.5%)
Sarcoma	25 (26.2%)
Solid tumor	38 (26.2%)
Protocol	
Chemotherapy	113 (77.9%)
Radiotherapy	21 (14.5%)
Surgery	42 (29.0%)
BMT	30 (20.7%)
Other	16 (11.0%)
WSD, M (SD)	82.7 (116.4)
Child's position	
1	49 (34.0%)
2	30 (20.8%)
3	22 (15.3%)
4+	43 (29.9%)

Abbreviations: BMT = Bone Marrow Transplant, WSD = Weeks Since Diagnosis.

### 4.2 Coping and Parental Distress Model

The analysis was conducted for the *PPSI Emotional and Physical* subscale and the *POMS Total* scale. Table 3 shows the results for both parental distress measures. Missing data were handled using listwise deletion, resulting in an analytic sample of 138 participants.

**TABLE 3:** Path coefficients for mediation models predicting parental distress.

Path	B	SE	*β*	95% CI	*p*‐value
	PPSI emotional and physical problems scale				
a	0.30	0.06	0.46	[0.18, 0.38]	< 0.001
b	−0.05	0.11	−0.04	[−0.29, 0.20]	0.660
c' (direct effect)	−0.10	0.07	−0.11	[−0.28, 0.06]	0.160
d1	−0.24	0.06	−0.29	[−0.35, −0.14]	< 0.001
d2	0.66	0.08	0.57	[0.49, 0.82]	< 0.001
ab (indirect effect)	−0.02	0.03	−0.02	[−0.08, 0.06]	0.660
d1d2 (indirect effect)	0.01	0.03	0.01	[−0.05, 0.08]	0.660
	POMS total scale				
a	0.29	0.05	0.45	[0.17, 0.37]	< 0.001
b	−0.03	0.11	−0.02	[−0.24, 0.19]	0.800
c' (direct effect)	−0.20	0.07	−0.22	[−0.36, −0.07]	< 0.001
d1	−0.24	0.06	−0.29	[−0.36, −0.14]	< 0.001
d2	0.66	0.08	0.58	[0.51, 0.80]	< 0.001
ab (indirect effect)	−0.00	0.03	−0.00	[−0.07, 0.05]	0.800
d1d2 (indirect effect)	−0.00	0.03	−0.00	[−0.05, 0.06]	0.800

*Note:* a path: Effect of coping repertoire on coping flexibility. b path: Effect of coping flexibility on parental distress. c' path: Direct effect of coping repertoire on parental distress. d1 path: Direct effect of avoidance‐focused coping strategy on coping flexibility. d2 path: Direct effect of avoidance‐focused coping strategy on parental distress. ab path: Indirect effect of coping repertoire on parental distress through coping flexibility. d1d2 path: Indirect effect of coping flexibility on parental distress through avoidance‐focused coping strategies. Confidence intervals and standard errors based on 5000 bootstrap re‐samples.

For the *PPSI Emotional and Physical Problems Scale,* the model explained 37.02% of the variance in parental distress [*Adjusted R* ^*2*^ = 0.36, *F* (3,133) = 26.06, *p* < 0.001], with avoidance‐focused coping strategy emerging as the only significant predictor, accounting for 28.85% of the total variance. Results also indicated that coping repertoire significantly and positively predicted coping flexibility. However, given the overwhelming contribution of avoidance‐focused coping strategy to the overall variance in parental distress, coping flexibility did not emerge as a significant predictor. Consequently, the indirect effect of coping repertoire on parental emotional distress through coping flexibility was not significant. The direct effect of coping repertoire on parental emotional distress was also not significant.

For the *POMS Total Score*, the model explained 43.01% of the variance in parental distress [*Adjusted R* ^*2*^ = 0.42, *F* (3,134) = 33.71, *p* < 0.001], with avoidance‐focused coping strategy emerging as the strongest predictor and accounting for 29.91% of the total variance. Results also indicated that, in accordance with the *PPSI Emotional and Physical Problems Scale*, coping flexibility did not significantly predict parental distress. Consequently, the indirect effect of coping repertoire on parental distress through coping flexibility was not significant. However, unlike the *PPSI* model, the direct effect of coping repertoire on the *POMS Total* Score was significant and accounted for an additional 3.82% of the total variance.

### 4.3 Additional Descriptive Analyses

As illustrated in Table 4, all avoidance‐focused coping techniques were significantly positively correlated with parental distress. While no specific problem‐, or emotion‐focused coping technique was associated with reduced distress, three emotion‐focused coping techniques (i.e., self‐distraction, venting, and religion) were significantly positively correlated with parental distress.

**TABLE 4:** Brief COPE techniques, classification by coping strategies and their bivariate correlation with distress outcomes.

Coping technique	PPSI emotional and physical scale	POMS total scale
Avoidance‐focused strategy		
Behavioral disengagement	0.32***	0.37***
Denial	0.44***	0.56***
Substance‐use	0.19*	0.16
Self‐blame	0.55***	0.50***
Emotion‐focused strategy		
Self‐distraction	0.27**	0.27***
Venting	0.52***	0.49***
Acceptance	−0.04	−0.03
Humor	−0.08	−0.16
Religion	0.22**	0.22***
Positive reframing	−0.12	−0.13
Social emotion‐support	0.01	−0.05
Problem‐focused strategy		
Social instrumental‐support	0.06	0.00
Planning	−0.08	−0.17*
Active coping	−0.05	−0.08

^*^ *p* < 0.05.

^**^ *p* < 0.01.

^***^ *p* < 0.001.

Table 5 shows Pearson correlations between the various coping and distress measures. As illustrated, greater use of avoidance‐focused coping strategy was significantly positively correlated with parental distress. Both coping repertoire and flexibility were significantly negatively correlated with parental distress. In addition, coping repertoire was significantly positively correlated with coping flexibility.

**TABLE 5:** Coping and distress measures: Bivariate correlations.

Measure	1	2	3	4	5	6	7	8	9	10
1. Avoidance‐focused strategy										
2. Emotion‐focused strategy	0.24**									
3. Problem‐focused strategy	−0.14	0.42***								
4. Coping repertoire: Positive	−0.08	0.44***	0.42***							
5. Flexibility: Abandonment	−0.26**	0.15	0.48***	0.38***						
6. Flexibility: Re‐coping	−0.31***	0.25**	0.53***	0.46***	0.51***					
7. Flexibility: Meta‐coping	−0.17*	0.28***	0.54***	0.43**	0.45***	0.66***				
8. Flexibility: Total	−0.33***	0.23**	0.58***	0.48***	0.88***	0.86***	0.63***			
9. PPSI: Emotional and physical scale	0.59***	0.23**	−0.03	−0.17*	−0.25**	−0.23**	−0.13	−0.28***		
10: PPSI: Total	0.37***	0.33***	0.12	−0.01	−0.08	0.05	0.11	−0.03	0.74***	
11: POMS: Total scale	0.62***	0.18*	−0.11	−0.28***	−0.27**	−0.30***	−0.16	−0.33***	0.83***	0.60***

^*^ *p* < 0.05.

^**^ *p* < 0.01.

^***^ *p* < 0.001.

## 5 Discussion

The current study examined the use of avoidance‐focused coping strategy, coping repertoire, and coping flexibility of parents of children with cancer and the individual and combined relationship between these variables and parental distress.

Our hypothesis was rejected. First, avoidance‐focused coping strategy was found to be positively correlated with parental distress. Parents who reported greater use of avoidance‐focused coping strategy, including behavioral disengagement, denial, substance use, and self‐blame techniques, reported higher levels of distress. A similar relationship between avoidance‐focused coping strategy and parental distress has been reported among various parent populations [18, 33, 34]. However, coping flexibility did not significantly mediate the correlation between coping repertoire and parental distress. Our results suggest that avoidance‐focused coping strategy is the most significant predictor of parental distress. Notably, when avoidance‐focused coping strategy was included in the regression model, the predictive effect of coping flexibility became non‐significant, despite its moderate negative bivariate correlation. This pattern suggests that while these constructs are related, avoidance‐focused coping strategy diminishes the potential benefits of flexibility. According to the dual‐process theory, *coping inflexibility* promotes engaging in a technique regarded as ineffective [19], while avoidance itself represents the strategy most detrimental to distress [13]. This may suggest that one cannot be flexible while simultaneously engaging in avoidance‐focused coping strategy.

Further analyses suggest that three emotion‐focused coping techniques—venting, self‐distraction, and religion—were also found to be positively related to parental distress. Studies suggest that venting may function similarly to rumination, involving repetitive focus on negative feelings and circumstances, which has been associated with greater psychological distress [35]. The mechanism underlying self‐distraction's association with distress remains unclear, with mixed findings in the literature. Self‐distraction may be adaptive when used temporarily to reduce overwhelming emotions, allowing individuals to regain composure before returning to address stressors directly [36]. However, when self‐distraction becomes a chronic or habitual avoidance technique, it is linked to higher levels of anxiety and depression, as it prevents facing the stressor or its emotional effect [36]. Finally, the cross‐sectional nature of the current study precludes any determination as to whether venting and self‐distraction are predictors or products of increased distress.

A positive correlation was found between religion as a coping technique and parental distress, a finding consistent with some studies [37], which may be explained through two complementary perspectives. First, the *Brief COPE's* religious coping measure has been noted for its unidimensional approach, consisting of only two items that fail to distinguish between positive and negative forms of religious coping [38]. To address this limitation, researchers recommend using the *Brief RCOPE* instead, which distinguishes between positive religious coping (e.g., seeking spiritual support, finding meaning) and negative religious coping (e.g., spiritual struggle, anger toward God) [38]. Second, religious coping effectiveness varies across different levels of religious commitment. Individuals with less religious commitment may be particularly vulnerable to spiritual struggle, as their faith‐based beliefs may be inadequate to account for traumatic experiences [39]. This suggests that religious coping may be beneficial for highly committed individuals while less helpful for those with weaker religious commitment.

### 5.1 Study Limitations

The results of the current study suggest that beyond the importance of coping repertoire and flexibility, the use of avoidance‐focused coping techniques is the most significant predictor of parental distress related to childhood cancer. While these findings provide valuable insight into parental coping and distress processes, several methodological limitations must be acknowledged when interpreting the results.

The current study's cross‐sectional design imposes significant limitations on causal interpretation. The mediation model presented in the current study assumes a specific temporal sequence according to which coping repertoire influences coping flexibility, which subsequently affects parental distress outcomes. This assumption requires strong theoretical justification, which we base on established coping theory [20]. Nevertheless, several alternative explanatory models could account for the observed relationships. For instance, a reverse mediation model might suggest that higher distress leads parents to develop greater coping flexibility as they desperately seek effective techniques, which then motivates them to expand their repertoire. Alternatively, reciprocal effects models would propose bidirectional relationships where distress influences parents' coping repertoire and flexibility, while these coping constructs simultaneously affect distress levels in an ongoing dynamic process. Moreover, while we conducted an a priori power analysis, our sample size may have been insufficient to detect smaller effect sizes that exist in the population. To enhance the robustness of our mediation findings, future longitudinal or experimental studies are needed. Such studies, employing larger and more diverse samples, could elucidate the temporal relationship between these constructs, while providing adequate power to detect subtle effects. Additionally, researchers could compare our proposed model with reverse mediation pathways, conduct sensitivity analyses to test model stability, and consider potential confounding variables that might create spurious associations between the constructs.

Beyond the mediation analysis, the correlational analyses presented in this study serve a primarily descriptive purpose, revealing bivariate associations between individual coping constructs and distress measures. These correlations do not control for potential confounding variables, assume linear relationships, and are sensitive to outliers. Therefore, they should not be interpreted as evidence of causal relationships. For example, our findings show that avoidance‐focused coping is associated with parental distress, but this relationship could operate in either direction: greater use of avoidance‐focused coping techniques may lead to greater parental distress, or alternatively, parents experiencing greater distress may rely more heavily on avoidance‐focused coping techniques.

Another potential limitation is the exclusive use of self‐report measures, which may introduce common method variance and potentially inflate the correlations between variables. Finally, a limitation of the current study relates to the *Brief COPE's* factor structure. A systematic review of 85 studies found that only 8 yielded the original 14‐factor structure, with identified factors ranging from 2 to 15 across studies [40]. The three‐factor structure (avoidance‐, emotion‐, and problem‐focused) employed in this study, while common in the literature, may not adequately reflect the complexity of coping processes and may potentially obscure specific techniques particularly relevant to parental distress.

### 5.2 Clinical Implications

The coping constructs studied in the current cross‐sectional study and the findings regarding their relationships to parental distress may have implications for clinical intervention. Future intervention studies should consider incorporating these constructs to determine how effective reducing avoidance‐focused coping, expanding repertoire, and enhancing flexibility might be in decreasing psychological distress among parents of children with cancer. A recent feasibility‐pilot study of a six‐session manualized intervention based on these constructs demonstrated its feasibility, with high levels of parental satisfaction and evidence of improved distress outcomes [41].

## 6 Conclusions

This cross‐sectional study suggests that among three coping constructs (i.e., avoidance‐focused coping, repertoire of problem‐ and emotion‐focused coping techniques, and coping flexibility), avoidance‐focused coping appears to be the strongest predictor of psychological distress among parents of children with cancer. Future studies should further examine the effect of these constructs on parental distress in larger and more diverse samples, using longitudinal and experimental study designs.

## Author Contributions

Oz Hamtzani and Michael J. Dolgin contributed equally to the conception, methodology, data collection, writing of the manuscript, and obtaining funding for the study. Talma Kushnir contributed through supervision, review, and editing of the manuscript. All authors have read and approved the manuscript.

## Ethics Statement

The study was approved by the ethics committees (Helsinki) of both Ariel University (AU‐SOC‐TK‐20220315) and the Edmond and Lily Safra Children's Hospital (SMC‐9041‐22).

## Conflicts of Interest

The authors declare no conflicts of interest.

## Data Availability

The data that support the findings of this study are available from the corresponding author upon reasonable request.
